# A nuclear phylogenomic tree of grasses (Poaceae) recovers current classification despite gene tree incongruence

**DOI:** 10.1111/nph.20263

**Published:** 2024-11-20

**Authors:** Grass Phylogeny Working Group III, Watchara Arthan, William J. Baker, Matthew D. Barrett, Russell L. Barrett, Jeffrey L. Bennetzen, Guillaume Besnard, Matheus E. Bianconi, Joanne L. Birch, Pilar Catalán, Wenli Chen, Maarten Christenhusz, Pascal‐Antoine Christin, Lynn G. Clark, J. Travis Columbus, Charlotte A. Couch, Darren M. Crayn, Gerrit Davidse, Soejatmi Dransfield, Luke T. Dunning, Melvin R. Duvall, Sarah Z. Ficinski, Amanda E. Fisher, Siri Fjellheim, Félix Forest, Lynn J. Gillespie, Jan Hackel, Thomas Haevermans, Trevor R. Hodkinson, Chien‐Hsun Huang, Weichen Huang, Aelys M. Humphreys, Richard W. Jobson, Canisius J. Kayombo, Elizabeth A. Kellogg, John M. Kimeu, Isabel Larridon, Rokiman Letsara, De‐Zhu Li, Jing‐Xia Liu, Ximena Londoño, Quentin W. R. Luke, Hong Ma, Terry D. Macfarlane, Olivier Maurin, Michael R. McKain, Todd G. B. McLay, Maria Fernanda Moreno‐Aguilar, Daniel J. Murphy, Olinirina P. Nanjarisoa, Guy E. Onjalalaina, Paul M. Peterson, Rivontsoa A. Rakotonasolo, Jacqueline Razanatsoa, Jeffery M. Saarela, Lalita Simpson, Neil W. Snow, Robert J. Soreng, Marc S. M. Sosef, E. John Thompson, Paweena Traiperm, G. Anthony Verboom, Maria S. Vorontsova, Neville G. Walsh, Jacob D. Washburn, Teera Watcharamongkol, Michelle Waycott, Cassiano A. D. Welker, Martin D. Xanthos, Nianhe Xia, Lin Zhang, Alexander Zizka, Fernando O. Zuloaga, Alexandre R. Zuntini

**Keywords:** Angiosperms353, genome, incongruence, phylogenomics, plastome, Poaceae, target capture, transcriptomics

## Abstract

- Grasses (Poaceae) comprise *c*. 11 800 species and are central to human livelihoods and terrestrial ecosystems. Knowing their relationships and evolutionary history is key to comparative research and crop breeding. Advances in genome‐scale sequencing allow for increased breadth and depth of phylogenomic analyses, making it possible to infer a new reference species tree of the family.
- We inferred a comprehensive species tree of grasses by combining new and published sequences for 331 nuclear genes from genome, transcriptome, target enrichment and shotgun data. Our 1153‐tip tree covers 79% of grass genera (including 21 genera sequenced for the first time) and all but two small tribes. We compared it to a newly inferred 910‐tip plastome tree.
- We recovered most of the tribes and subfamilies previously established, despite pervasive incongruence among nuclear gene trees. The early diversification of the PACMAD clade could represent a hard polytomy. Gene tree–species tree reconciliation suggests that reticulation events occurred repeatedly. Nuclear–plastome incongruence is rare, with very few cases of supported conflict.
- We provide a robust framework for the grass tree of life to support research on grass evolution, including modes of reticulation, and genetic diversity for sustainable agriculture.

## Introduction

With almost 11 800 species in 791 genera (Soreng *et al*., 2022), grasses (Poaceae) are among the largest plant families and one of the most important for humans. Grasses include the primary food crops rice, maize and wheat, sources of fibre and building materials such as reed and bamboo, and biofuel crops such as sugarcane and switchgrass. Much of the global land surface is covered by grass‐dominated ecosystems, where grasses impact productivity, nutrient cycling and vegetation structure by mediating fire and herbivory (Edwards *et al*., 2010; Bond, 2016). Grasses are also overrepresented among the world's most damaging agricultural weeds (Holm *et al*., 1977) and invasive plants (Linder *et al*., 2018). Understanding functional diversification, adaptation and novel crop breeding in this important plant group requires a solid understanding of its evolutionary relationships.

Efforts to uncover the phylogenetic history of grasses have tracked the development of new technology and analytical tools, beginning with cladistic analysis of morphology (e.g. Campbell & Kellogg, 1987). Almost as soon as nucleotide sequencing became possible, it was used to investigate grasses (rRNA sequencing, Hamby & Zimmer, 1988, and chloroplast DNA, Clark *et al*., 1995), and the results interpreted in the light of known morphology and classification. Hundreds of papers have been published since using nucleic acids, most recently DNA, to assess grass phylogeny at all taxonomic levels and assembling information from all three genomes in the cell (plastid, mitochondrial, and nuclear). These efforts have been punctuated by two major phylogenetic analyses, Grass Phylogeny Working Group I (GPWG, 2001) and GPWG II (2012), and family‐wide classifications (Kellogg, 2015; Soreng *et al*., 2022) were enabled by these and many other detailed phylogenetic analyses.

The major outlines of grass phylogeny have now been known for several decades and corroborated by accumulating data, with major lineages recognised as subfamilies (Kellogg, 2015; Soreng *et al*., 2022). The earliest divergences in the grass family gave rise to three successive lineages, Anomochlooideae, Pharoideae, and Puelioideae, each comprising just a few species. After the divergence of those three, however, the remaining grasses gave rise to two sister lineages, known as BOP and PACMAD, each of which became a species‐rich clade with several robust subclades. This sturdy phylogenetic framework is reflected in a strong subfamilial classification, with subfamilies divided into equally robust tribes. Attention in recent years has largely shifted to relationships of tribes, subtribes, and genera.

Reticulate evolution is common in the grasses. Allopolyploidy is widespread in the family, particularly among closely related species and genera, with as many as 80% of species estimated to be of recent polyploid origin (Stebbins, 1985). The textbook example is bread wheat (*Triticum aestivum*) and its ruderal annual ancestors, the history of which was determined in the first part of the 20^th^ century using cytogenetic tools (Kihara, 1982; Tsunewaki, 2018). Nucleotide sequence data have verified the hybrid origin of wheat and gone on to show that reticulate evolution is the norm in the entire tribe Triticeae (Feldman & Levy, 2023; Mason‐Gamer & White, 2024). We have also learned that three of the four major clades of Bambusoideae are of allopolyploid origin (Triplett *et al*., 2014; Guo *et al*., 2019; Chalopin *et al*., 2021; Ma *et al*., 2024), as are at least one third of the species in Andropogoneae (Estep *et al*., 2014). Large‐scale lateral gene transfer has also been demonstrated in *Alloteropsis semialata* (Dunning *et al*., 2019) and for a number of genomes across the family (Hibdige *et al*., 2021), although it remains unclear how common such genetic exchanges are. Network‐like reticulations are therefore expected throughout Poaceae.

Data relevant to grass phylogeny continue to accumulate in the genomic era, but in an uneven pattern. Major recent studies have inferred family trees based on the plastid genome (Saarela *et al*., 2018; Gallaher *et al*., 2022; Hu *et al*., 2023) or large parts of the nuclear genome (Huang *et al*., 2022). In addition, a wealth of full‐genome assemblies is now available for grasses, mainly for groups that have been studied intensively, such as major crops and their congeners including rice (Wang & Han, 2022), maize (Hufford *et al*., 2021), wheat (Walkowiak *et al*., 2020) and sugarcane (Healey *et al*., 2024), among many others. At the same time, some genera and many species remain virtually unknown beyond a scientific name and general morphology. While the poorly known taxa may be represented in major herbaria, fresh material can be hard to obtain, weakening attempts to fully sample the grass tree of life with phylogenomic technologies.

Fortunately, we are now experiencing the confluence of: (1) global sources of diversity data including plant specimens held in herbaria world‐wide, (2) widespread use of short‐read sequencing that can accommodate even fragmented DNA, (3) analytical tools for assembling and interpreting massive amounts of sequence data, and (4) technical tools for efficient sequencing, such as target capture. For example, the development of a universal probe set for flowering plants, Angiosperms353 (Johnson *et al*., 2019; Baker *et al*., 2021), has enabled initiatives to sequence all angiosperm plant genera (Baker *et al*., 2022; Zuntini *et al*., 2024) or entire continental floras such as that of Australia (https://www.genomicsforaustralianplants.com/). It became apparent that an updated synthesis of existing and new data for grasses, similar to the previous Grass Phylogeny Working Group efforts (GPWG, 2001; GPWG II, 2012), would be timely and make possible a phylogeny that incorporates representatives of most of the 791 genera of the family using genome‐scale data. In the process, we will gain a broader assessment of congruence among nuclear gene histories, including insights on the frequency and impact of incomplete lineage sorting (ILS) and reticulation.

Accordingly, here we present the most comprehensive nuclear phylogenomic tree of the grass family to date. Via a large community effort, we maximised taxon sampling by combining whole‐genome, transcriptome, target capture and shotgun datasets. Based on the Angiosperms353 gene set, we inferred a nuclear multigene species tree using a coalescent‐based method that accounts for incongruence due to ILS and uses information from multicopy gene trees. We also inferred a plastome tree and tested for incongruence between plastome and nuclear trees. Finally, we used gene tree–species tree reconciliation analyses to explore the signal for reticulation in the nuclear data.

## Materials and Methods

### Datasets and species sampling

Drawing from a combined effort of the Poaceae research community, we leveraged five diverse sets of genomic data (see full accession table in the data repository, doi: 10.5281/zenodo.10996136). We deployed a set of automated filters and repeated expert input from the group to remove duplicates, samples with insufficient data, and potentially misidentified accessions. The final set of accessions included:

1. 450 Illumina target capture read accessions enriched with the Angiosperms353 probe set (Johnson *et al*., 2019), generated as part of the ‘Genomics for Australian Plants’ (GAP) and ‘Plant and Fungal Trees of Life’ (PAFTOL; Baker *et al*., 2022) initiatives as well as a project focused on Loliinae grasses (P. Catalán *et al*., unpublished data). Sampling focused on genera without existing nuclear or plastome genomic data.
2. 295 Illumina shotgun, whole‐genome sequencing accessions, of which 204 are ‘genome skims’ with a sequencing depth < 5× estimated for our target gene set (to be described later). Of these shotgun accessions, many had been used in previous studies for the assembly of plastid genomes (see accession table).
3. 17 Illumina target capture read accessions enriched in 122 nuclear loci (different from Angiosperms353) that were previously used in a phylogenetic study of the subfamily Chloridoideae (Fisher *et al*., 2016). These are treated here like the shotgun datasets.
4. 343 assembled transcriptomes from two recent Poaceae studies (331 samples; Huang *et al*., 2022; Zhang *et al*., 2022) and the 1KP initiative (12 samples, One Thousand Plant Transcriptomes Initiative, 2019).
5. 48 assembled and annotated genome sequences from Phytozome v.13, Ensembl Plants, or other sources.

Angiosperms353 target capture data were generated by the PAFTOL project following the protocols of Baker *et al*. (2022). Methods varied for the other contributed datasets (details in accession table and Supporting Information Methods S1). Leaves were sampled mostly from herbarium specimens, although silica dried material was used in some cases. Sampling was iteratively refined using expert input from the working group to remove accessions with unclear identity and duplicates per species (retaining the highest‐coverage accession, that is genome > transcriptome > target capture > shotgun). Species names were harmonised using the World Checklist of Vascular Plants (Govaerts *et al*., 2021) as well as expertise from our working group.

### Grass‐specific Angiosperms353 reference dataset

Before sequence assembly from target capture and shotgun datasets, we produced a Poaceae‐specific set of reference Angiosperms353 sequences to improve recovery and account for grass‐wide gene duplications. This grass‐specific reference dataset consists of coding sequences (CDS) extracted from published genomes and transcriptomes of 60 species, representing seven of the 12 grass subfamilies and including an available genome sequence from the sister group Ecdeiocoleaceae–Joinvilleaceae (*Joinvillea ascendens* Gaudich. ex Brongn. & Gris). First, CDS of the Angiosperms353 homologs were extracted from the reference genomes and transcriptomes using the tblastn tool of Blast+ v.2.2.29 (Camacho *et al*., 2009), with the original Angiosperms353 probe set used as protein queries (e‐value ≤ 10^−3^). To reduce false positives, only hits with alignments > 65% of the query length and sequence identity > 60% were retained. This filtered homolog set was then sorted into orthogroups using Orthofinder v.2.5.2 (Emms & Kelly, 2019), with the MSA mode using Mafft v.7.481 (Katoh & Standley, 2013) as the sequence aligner, and FastTree v.2.1.11 (Price *et al*., 2010) to generate gene trees, using default parameters in each case.

Using the phylogenetic hierarchical method of Orthofinder, we extracted orthogroups at the level of the most recent common ancestor of the BOP–PACMAD clade, the crown group which covers > 99% of grass species and most available reference genomes. Two of the original Angiosperms353 markers (g5422 and g6924) were not detected in any of the reference genomes or transcriptomes and were therefore not used. Five other markers were duplicated before the BOP–PACMAD split (g4527, g5434, g5945, g5950 and g7024); these duplicates were therefore treated as separate markers in our analyses. For these five duplicated genes, homologs of the three reference samples representing subfamily Anomochlooideae, sister to all other Poaceae, and the outgroup Joinvilleaceae were subsequently added to each of the two corresponding orthogroups. This initial reference dataset was then curated to remove nonhomologous sequences and potential pseudogenes (see Methods S1). The final reference dataset consisted of 356 orthogroups, and encompassed all homologous sequences of the 60 reference species, including paralogs from lineage‐specific duplications within each orthogroup. Note that three of the markers (g5328, g5922 and g6128) were removed before phylogenetic analysis on the basis that they contained regions of low complexity in their sequences, which resulted in low‐quality assemblies (to be described later) as revealed by preliminary analyses.

### Angiosperms353 sequence assembly

The orthogroup dataset was used as a reference for sequence assembly using HybPiper v.1.3.1 (Johnson *et al*., 2016). Illumina reads were initially trimmed using Trimmomatic v.0.38 (Bolger *et al*., 2014) to remove adapters, low‐quality bases and short reads (SLIDINGWINDOW:4:20, MINLEN:40). Sequences were assembled using the Burrows‐Wheeler Alignment tool (Bwa, Li & Durbin, 2009) with default parameters, except the coverage cut‐off level, which was reduced to 4× for the target capture datasets, and to 1× for shotgun accessions due to the low‐sequencing depth of a subset of samples. Given the low number of markers recovered for most shotgun accessions, we used a custom assembly strategy optimised for the assembly of sequences from low‐coverage datasets (explained below). When a sequence was assembled by both HybPiper and the custom method, only the longest assembly was retained.

The custom assembly strategy consisted of a mapping‐consensus pipeline modified from Olofsson *et al*. (2019) and Bianconi *et al*. (2020) to support the assembly of paralogs (Fig. S1). First, filtered reads were mapped to the orthogroup reference dataset using Bowtie2 v.2.5.3 (Langmead & Salzberg, 2012) with the sensitive‐local mode and reporting all alignments. Then, for each orthogroup, the reference sequence with the most bases covered was identified and included along with its paralogs (i.e. homeologs or paralogs from lineage‐specific duplications) in a second, accession‐specific reference dataset. This reduced the reference dataset to a single species per orthogroup, which allowed subsequent read mapping refinement, and simplified downstream processing. Read mapping was then repeated on this accession‐specific reference using the parameters described above, and the resulting read alignments were converted into majority consensus sequences using Samtools v.1.19.2 (Li *et al*., 2009; *consensus* function, *‐‐min‐depth 1 ‐‐het‐fract 1 ‐‐call‐fract 0.5*). Only consensus sequences longer than 200 bp were retained for downstream analysis. Cases of multiple assemblies within a given orthogroup were treated as potential paralogs and subsequently inspected to remove spurious assemblies. First, identical assemblies (full length or partial) were removed using SeqKit v.2.7.0 (Shen *et al*., 2016) and Cd‐Hit v.4.8.1 (Fu *et al*., 2012). If multiple assemblies remained for a given orthogroup, these were aligned together with the reference sequences used for their assembly using Mafft. A phylogenetic tree was then estimated using Iq‐Tree v.2.1.3 (Minh *et al*., 2020; substitution model HKY) and rooted on the longest branch. Only assemblies that formed a monophyletic group with their corresponding references were validated as paralogs and retained for downstream analyses. In all other cases, only the longest assembly was retained. Steps that involved tree manipulation were implemented using Newick Utilities v.1.6 (Junier & Zdobnov, 2010). Note that this approach only recovers paralogs from duplication events that are shared with one of the reference species, so that paralog recovery is expected to be limited in groups that are not represented in the reference dataset. In such cases, paralogs from lineage‐specific duplications are expected to be collapsed into single sequences, with differences coded as ambiguities. While such chimeric sequences might add noise to gene tree estimation, particularly in the relationships among accessions that share the duplicates, this is an intrinsic limitation of short‐read data, which cannot be fully overcome by our custom assembly strategy or HybPiper, although we expect their impact to be reduced due to the filters that are in place.

The performance of the custom assembly strategy was evaluated by reconstructing the Angiosperms353 sequences of two species from the reference dataset for which high‐quality genomes are available (*Brachypodium distachyon* (L.) P.Beauv. and *Oryza sativa* L.). For this, shotgun read datasets for these species were downloaded from the NCBI SRA database (accessions SRR891794 and SRR24031307) and subsampled to create four sets with varying sequencing depths (1, 5, 10 and 20×). Sequences were then assembled using our pipeline and compared to the sequences extracted from the reference genomes to assess the effect of sequencing depth on sequence completeness and identity, and on the recall of paralogs (Figs S2, S3).

### Extracting Angiosperms353 homologs from transcriptomes

To identify Angiosperms353 homologs in the transcriptome accessions, we performed a BLASTn search with the orthogroup reference dataset as query (e‐value ≤ 10^−3^), and retained only hits with alignments covering > 50% of the query length and nucleotide identity > 70% for phylogenetic analysis. For the orthogroups corresponding to Angiosperms353 markers that were duplicated before the BOP–PACMAD split (as mentioned in the previous section), a BLASTn search was conducted and filtered as above, except that the query included the reference sequences of the two paralogous orthogroups. The putative homologous hits were then sorted into their corresponding orthogroups by aligning each hit with the query sequences using Mafft, and estimating a tree using Iq‐Tree. The hit was then assigned to one of the orthogroups based on the clade in which it was nested in the tree.

### Nuclear tree inference

We used all the recovered sequences, including paralogs from lineage‐specific duplications within loci, for inferring a species tree using a coalescent‐based approach that accounts for paralogy, which has been shown to improve species tree estimation and vastly increase the data available for analysis (Smith & Hahn, 2021; Yan *et al*., 2021; Smith *et al*., 2022). Gene alignments were generated in a two‐step approach. First, the reference sequences were aligned using Mafft (*‐‐maxiterate* = 100) to generate a backbone alignment per gene. Then, gene assemblies of shotgun, target capture and transcriptome accessions were aligned one by one using the options *‐‐addfragments* and *‐‐keeplength* to improve the quality of the alignment of partially assembled sequences. Alignments were trimmed using trimAl v.1.4 (Capella‐Gutiérrez *et al*., 2009) to remove columns with 90% or more missing data (*‐gt* 0.1), and individual sequences shorter than 200 bp were removed from the trimmed alignments. To reduce uncertainty in tree estimation due to insufficient data, we discarded gene alignments with a total length of < 500 bp after trimming. Finally, to further reduce the impact of missing data, only accessions with at least 50% of the total gene set were kept for analysis. The resulting dataset consisted of 1153 accessions and 331 gene alignments. Gene trees were then inferred using RAxML v.8.2.12 (Stamatakis, 2014) with 100 rapid bootstrap pseudoreplicates. To strike a balance between computation time and modelling rate heterogeneity adequately, we used a GTR substitution model with a CAT rate heterogeneity approximation (25 rate categories; Stamatakis, 2006) across each alignment. Abnormally long branches that significantly inflated tree diameter were detected using TreeShrink v.1.3.9 (Mai & Mirarab, 2018), with the false positive rate set to 0.1 (option *‐q*). These were then removed from the alignments, and the phylogenetic analysis was repeated. Branch support in gene trees was measured using transfer bootstrap expectation (TBE), which provides a gradual, rather than a presence–absence, measure of support and is more robust to rogue tips in large trees compared to classical Felsenstein bootstrap proportion (Lemoine *et al*., 2018).

A multigene coalescent species tree was inferred using the resulting 331 gene trees with Astral‐Pro3 v.1.17.3.5 (Zhang *et al*., 2020). As measures of branch support and conflict in the species tree, we used the Quartet Concordance (QC) and Quartet Differential (QD) metrics described by Pease *et al*. (2018). They were calculated from the paralogue‐weighted proportions of gene trees supporting each of the three possible quartets around a branch, as reported by Astral‐Pro (R script ‘quartet_metrics.R’ in the data repository). Following Pease *et al*., we interpret QC values > 0.2 as strong support for one preferred quartet and values between 0 and 0.2 as indicating conflict between gene trees (the species tree already shows the majority quartets, so values cannot be < 0). QD will be 1 when the second and third alternative quartets are recovered with equal frequency, as expected under ILS, especially when QC indicates conflict with the first quartet. When conflict is skewed to only two preferred alternatives in total at a branch, for example under introgression or hybridisation, QD will approach zero.

We evaluated tree stability across two additional data filtering strategies. In the first, the effect of missing data was assessed by increasing the alignment trimming threshold and removing columns with > 50% missing data (all 1153 samples retained). In the second filtered set, the same filtering strategy of the main dataset was used, but to be sure that our novel assembly methods were not biasing the results, we tested the impact of omitting the shotgun sequences altogether (841 tips retained; i.e. only accessions from target capture, transcriptome and complete genome sources). We compared support and conflict in the multigene coalescent tree across the three filtered sets using as metrics QC, QD and the proportions of gene trees informative per branch. We counted the number of matching branches (based on tip sets) of the additionally filtered sets compared to the main tree and summarised support and conflict at these branches. We also calculated a measure of gene tree–species tree distance (Clustering Information Distance; Smith, 2020) using the TreeDist R package v.2.7 (Smith, 2019); this required keeping only one paralog, chosen randomly, for multicopy accessions in the gene trees.

### Gene tree–species tree reconciliation

We investigated the evidence for reticulations, whether from hybridisation, introgression or lateral transfers. We used gene tree–species tree reconciliation under the maximum likelihood implementation of a duplication–transfer–loss model (UndatedDTL) in GeneRax v.2.0.1 (Morel *et al*., 2020). This is not equivalent to a full, computationally expensive phylogenetic network analysis (such as PhyloNet, Wen *et al*., 2018) but instead assumes a true bifurcating species tree, which hugely constrains the search space and makes the analysis amenable to our data. Note that apparent transfers may also reflect ILS, which is not modelled by GeneRax, but we expected this to be limited to lineages branching in short succession. Running the analysis for the whole dataset was not feasible, so we performed a tribe‐level reconciliation, where the species tree was collapsed to tribes, with gene trees matched to these tribes. We ran additional reconciliation analyses for three clades of economic importance and with well documented reticulation histories: subfamily Bambusoideae (bamboos), tribe Andropogoneae (maize, sorghum and relatives), and Triticeae (wheat and relatives).

From the gene trees, GeneRax infers, in addition to duplications and losses, gene transfers between two branches of the species tree. We summarised these transfers on the species tree using custom R scripts (see data repository). Because an apparent transfer may also be an artefact of a poorly supported gene tree, we considered that a transfer between two branches had to be supported by at least five gene trees to indicate possible reticulation. Note that in the case of the tribe‐level tree, this number of transfers combines gene tree tips from all species within a tribe. Transfers to/from the root were excluded (as they might involve any branch outside the ingroup that was not sampled). We highlighted the most frequent transfers as those with the top 10% quantile counts per species tree. We also evaluated, for each reticulate connection, if transfer counts were skewed in one direction by highlighting those with > 50% proportional difference between counts in either direction.

### Plastome sequence assembly and tree inference

To compare the nuclear topology with the plastome topology, we inferred a 910‐tip tree using the sequences of 70 coding plastome regions and the *trnL–trnF* intergenic region. We retrieved the 520 assembled plastome sequences that were already publicly available, representing in most cases shotgun accessions in the nuclear analysis, and sequences from the same species if the same accession was not available (see metadata table in data repository). New plastome CDS were assembled from shotgun and Angiosperms353 Illumina data using getOrganelle v.1.7.5 (Jin *et al*., 2020) with default kmer settings for SPAdes (21, 45, 65, 85, 105) and 15 maximum extension rounds. We used a well‐annotated plastome sequence (*Digitaria exilis* (Kippist) Stapf, INSDC accession KJ513091.1) as seed for assembly. Plastome assemblies were annotated using GeSeq (Tillich *et al*., 2017). The target sequences were then recovered from the full or partial assemblies via Blast, with the *D. exilis* sequences as queries. Assemblies per sample were selected to cover at least 25% of the reference length for at least five genes or intergenic regions. Sequences were aligned per gene using Mafft, alignment columns containing large proportions of gaps were trimmed using the automated algorithm of trimAl v.1.4.15 and all gene alignments concatenated using Amas (Borowiec, 2016). After this step, accessions with 95% or more missing sites were removed, leaving the final 910 accessions. A maximum likelihood tree was then inferred using RAxML v.8.2.12 with a GTR‐CAT model and 100 rapid bootstrap pseudoreplicates.

To measure to what degree nuclear relationships were supported by the plastome analysis, we mapped quartet support from 100 plastome bootstrap trees on the nuclear tree using ASTRAL‐Pro, after reducing both sets of trees to 751 tips we could match by accession, or, if the same accession was not available, by species. From the bootstrap frequencies per quartet, we calculated QC, which here will be 1 if the plastome tree supports the same quartet, and −1 if the plastome tree strongly supports an alternative quartet. We also tested if nuclear–plastome conflict tends to affect branches where there is also conflicting signal within the nuclear genome by correlating QC calculated from nuclear gene trees with QC calculated from plastome bootstrap trees.

## Results

### Nuclear reference dataset and genomic data

We compiled a grass‐specific reference dataset for the assembly of 356 nuclear genes, available in the data repository (file ‘target_Ang353_sequences_grasses.zip’, doi: 10.5281/zenodo.10996136). These genes were then extracted from genome and transcriptome sequences, and assembled from target capture and shotgun data.

The final dataset used for phylogenetic analysis consisted of 1153 accessions and 331 genes. Taxon occupancy was above 70% in 95% of all genes, and the number of genes recovered per accession ranged from 166 to 331 (median = 308). Median gene recovery was highest in shotgun accessions (98%), followed by transcriptomes (93%), target capture (92%) and genomes (91%) (Fig. S2a; Table S1). The lower gene recovery in genomes was a result of the stringent filters applied to prevent the incorporation of deep paralogs and nonhomologous sequences into the grass‐specific reference dataset (see the Materials and Methods section and Methods S1), which occurred at the expense of discarding some true orthologues. Among shotgun accessions, gene recovery was correlated with sequencing depth, although sequencing depth as low as 1× was in most cases sufficient to recover sequences (> 200 bp) for more than 90% of all genes (Fig. S2b). Nonetheless, as expected, mean sequence completeness was higher among genome and transcriptome accessions (median = 85% and 83%) than in shotgun and target capture accessions (median = 63 and 60%; Fig. S2c). We were able to recover at least 80% of the Angiosperm353 genes (with sequences on average 49% complete) for the 17 target capture samples that had been originally enriched for 177 different nuclear loci (Fisher *et al*., 2016).

Paralogs from lineage‐specific duplications were present in all 331 genes, and the median number of species with paralogs across genes was 31 (min = 4, max = 138; Table S2). Paralogs were more frequent in accessions represented by complete genomes, with on average 30% of the accessions having paralogs in each gene, followed by shotgun (4%), target capture (1.5%) and transcriptomes (0.5%; Fig. S3). In shotgun datasets, the number of genes with paralogs varied among accessions, and in some cases it was correlated with sequencing depth (Figs S4, S5), although this pattern was not consistent in the simulated datasets (Figs S6, S7). Such an overall low paralog recovery is in part explained by the filtering strategy of the custom assembly method, which retained only 17% of the putative paralogous sequences assembled (Table S2).

Increasing filtering stringency overall reduced missing data, at the expense of reducing the number of tips and/or alignment length (Table S1). For example, mean alignment completeness was increased from 73% to 79% by increasing alignment‐trimming stringency, while reducing mean alignment length from 1160 to 864. Likewise, by removing the 312 shotgun accessions, mean alignment completeness was only slightly increased to 76% (Table S1).

### Nuclear genome phylogeny

**Fig. 1:**
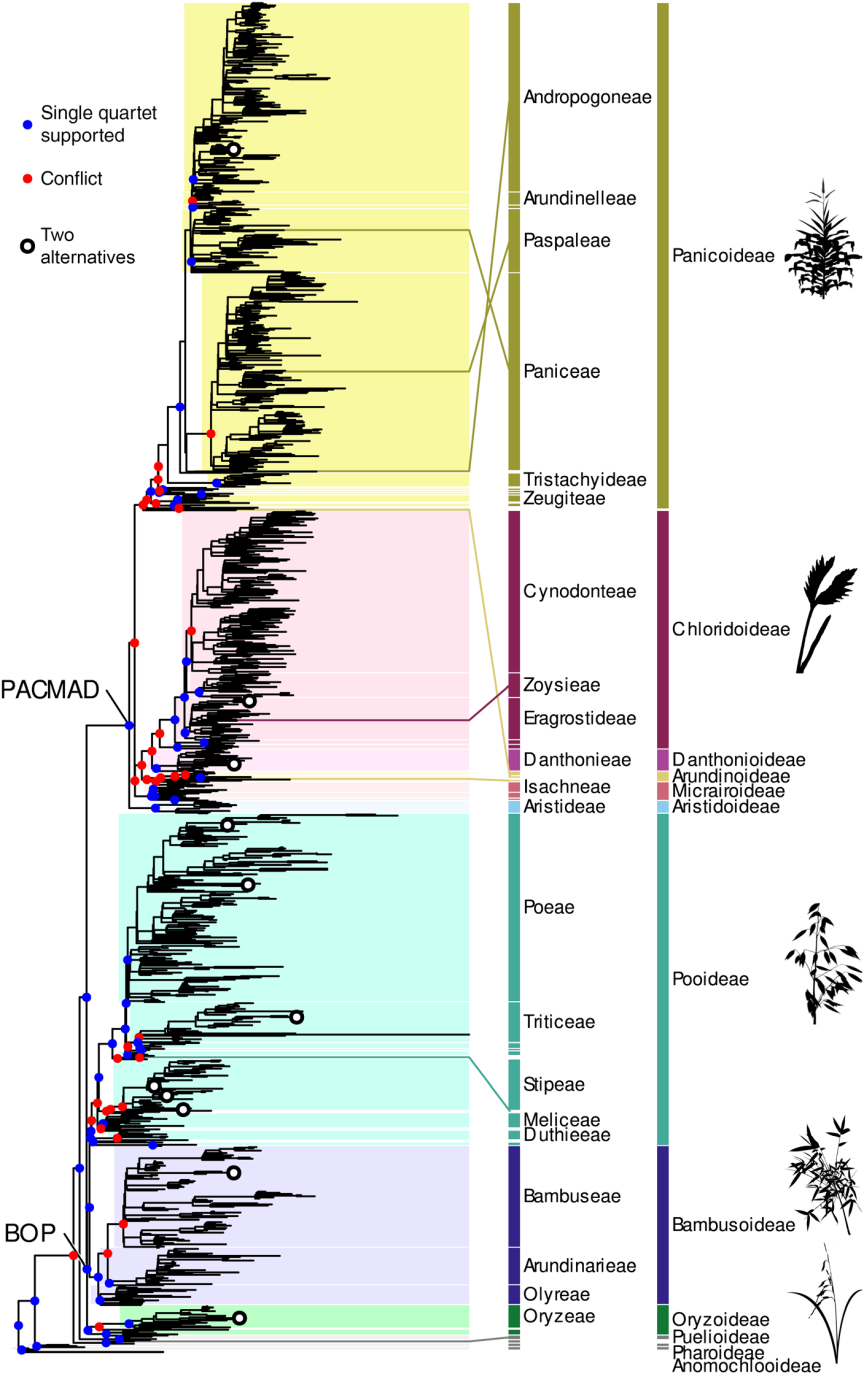
Phylogeny of 1153 Poaceae accessions inferred from 331 nuclear genes, including paralogs, using a multispecies coalescent approach. Closed dots indicate support or conflict on branches above tribe level based on the Quartet Concordance (QC) and Quartet Differential (QD) metrics, with blue dots indicating support for the quartet shown (QC > 0.2) and red dots indicating conflicting alternatives (QC ≤ 0.2). Open circles indicate supported conflict among nuclear gene trees at 11 branches, where two alternative quartet configurations are supported (QC ≤ 0.2 and QD < 0.5). Subfamilies and larger tribes (abbreviated) are labelled according to the most recent Poaceae classification (Soreng *et al*., 2022). The coloured lines link taxonomic outliers at tribe to subfamily level to their nominal taxa. Silhouettes show representatives for large subfamilies (from top): Maize or corn, *Zea mays* (Panicoideae); *Dactyloctenium radulans* (Chloridoideae); oat, *Avena sativa* (Pooideae); *Bambusa textilis* (Bambudoideae); rice, *Oryza sativa* (Oryzoideae). See Supporting Information Fig. S8 for a detailed version of the tree.

Our 1153‐tip species tree recovered almost all subfamilies and tribes of Poaceae but points to frequent gene tree incongruence (Fig. 1; see also detailed plot of the tree broken down into subclades in Fig. S8). Of the internal branches, only just above one quarter (314 of 1151) had one strongly preferred quartet configuration (QC > 0.2). Clades with conflicting signals above tribe level (QC ≤ 0.2) include BOP + PACMAD + *Puelia* + *Guaduella*, subfamily Panicoideae, and several divergences between subfamilies in the PACMAD clade and in subfamily Pooideae. The distribution of gene tree conflict, with QC values skewed towards zero, remained almost unchanged when the dataset was filtered more stringently (Fig. S9a), despite the high resolution in gene trees (Fig. S9g). The distribution of QD was strongly skewed towards 1, that is the second and third alternatives for each quartet had mostly similar frequencies, matching expectations under frequent ILS. QD may be distorted when one quartet is strongly preferred and frequencies of the second and third quartet are low (high QC), but the QD skew towards 1 becomes even clearer when looking only at highly conflicted branches (QC ≤ 0.2, Fig. S9b). It also holds under more stringent gene tree filtering, suggesting indeed ILS rather than the effect of poorly supported, randomly resolving gene trees. Support for two alternative resolutions, expected under hybridisation or introgression, was rare, with only 11 instances where branches showed strong conflict (QC ≤ 0.2) and a > 50% skew in the frequencies of the second and third quartet (QD < 0.5). The median number of gene trees informative about a given branch/quartet (from 331) was 202 (61%), with a range from 72 to 290 (22–88%) (Fig. S9c). Filtering the combined dataset more stringently had negligible effects on species tree support or conflict (Fig. S9), both overall (Fig. S9a–c) and in direct comparison of matching branches (Fig. S9d–f). More stringent filtering had only slight effects on gene tree support (slight increase, Fig. S9g) and gene tree distance from the species tree (slight decrease, Fig. S9h).

We compared our tree to the most recent Poaceae classification (Soreng *et al*., 2022). The 1153 accessions correspond to 1133 accepted species, covering all but two (Anomochloeae and Streptogyneae) of the accepted tribes and 621 (79%) of the 791 genera. Twenty‐one genera were sequenced for the first time: *Asthenochloa* Buse, *Bhidea* Stapf ex Bor, × *Cynochloris* Clifford & Everist, *Dilophotriche* (C.E.Hubb.) Jacq.‐Fél., *Fimbribambusa* Widjaja, *Ekmanochloa* Hitchc., *Kaokochloa* De Winter, *Hydrothauma* C.E.Hubb, *Mniochloa* Chase, *Parabambusa* Widjaja, *Pinga* Widjaja, *Pogonachne* Bor, *Pommereulla* L.f., *Ratzeburgia* Kunth, *Ruhooglandia* S.Dransf. & K.M.Wong, *Spathia* Ewart, *Suddia* Renvoize, *Taeniorhachis* Cope, *Thedachloa* S.W.L.Jacobs, *Thyridachne* C.E.Hubb., and *Trilobachne* Schenck ex Henrard. All subfamilies were recovered as monophyletic, except for the early‐diverging Puelioideae, which is paraphyletic, with its two genera *Guaduella* and *Puelia* forming separate lineages, as also noted by Huang *et al*. (2022). In Panicoideae, a clade comprising all accepted tribes is supported, but the branch subtending this clade plus *Alloeochaete* and *Dichaetaria*, only recently transferred from Arundinoideae (Teisher *et al*., 2017; Soreng *et al*., 2022) shows gene tree conflict. We found six further taxonomic discrepancies at tribe or subfamily level where tip positions did not match the taxonomy (Fig. 1; Table 1), and further cases of nonmonophyly at the subtribe level (see detailed tree in Fig. S8).

**Table 1:** Taxonomic discrepancies in the nuclear tree at subfamily to tribe level.

Genus/species	Nominal taxon	Nuclear tree position	Plastome tree position
*Amphipogon strictus* R.Br.	Arundinoideae: Arundineae	Sister to Crinipedeae + Molinieae	In Arundinoideae: Arundineae
*Baptorachis foliacea* (Clayton) Clayton*	Paspaleae	Paniceae: Anthephorinae	Paniceae: Anthephorinae
*Chaetium festucoides* Nees*	Panicoideae: Paniceae	In Paspalinae, sister to Streptostachys	Not included
*Guaduella* Franch.	Puelioideae	Sister to (*Puelia* + BOP + PACMAD)	Sister to *Puelia*
*Neomolinia* Honda & Sakisaka*	Pooideae: Diarrheneae	Sister to Brachypodieae + Triticodae + Poodae	Not analysed (sister to Diarrhena in Gallaher *et al*., 2022)
*Ratzeburgia pulcherrima* Kunth*	Panicoideae: Andropogoneae: Ratzeburginae	Sister to Paniceae	Not included
*Sporobolus subtilis* Kunth.	Chloridoideae: Zoysieae	In Eragrostideae, in *Eragrostis*	In Eragrostideae, in *Eragrostis*
*Styppeiochloa hitchcockii* (A.Camus) Cope	Arundinoideae: Crinipedeae	Sister to Panicoideae	In Arundinoideae: Crinipedeae

Taxa listed here will need follow‐up studies to validate their placement. An asterisk (*) denotes genera whose type species was sampled.

Two accessions in surprising, isolated positions within Panicoideae (*Styppeiochloa hitchcockii* and *Ratzeburgia pulcherrima*, Table 1) passed all quality filtering steps. Individual gene tree plots suggested unstable positions, but no clear indication of a laboratory mix‐up or contamination. Because no prior DNA data are available for these species, we retained them in the analysis but emphasise the need for further validation with independent samples.

### Gene tree–species tree reconciliation

**Fig. 2:**
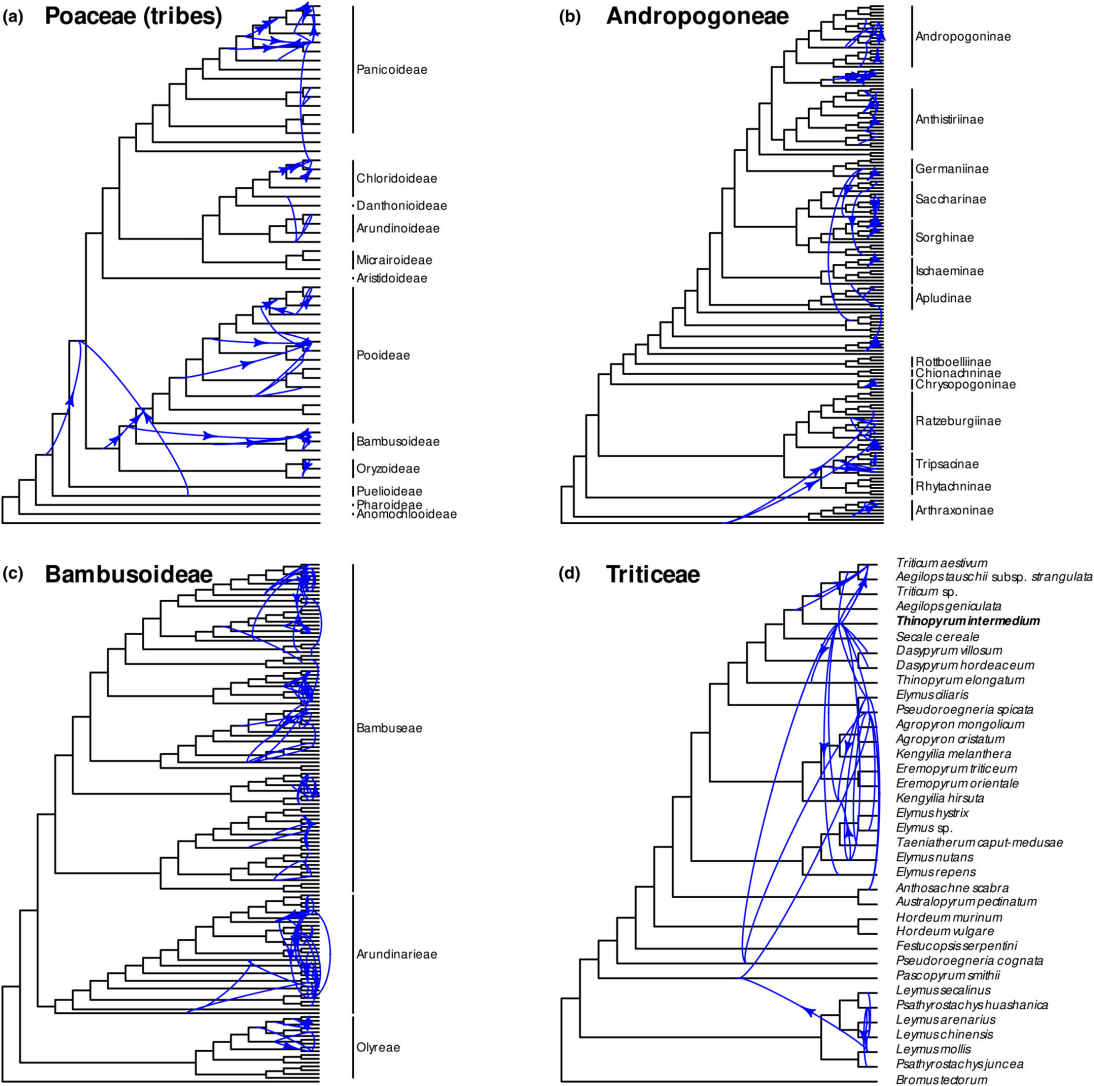
Nuclear gene reticulation in the grass family. For selected subgroups, the 331 gene trees were reconciled with the species tree under a duplication–transfer–loss model. Blue curves represent transfer events between two branches inferred at least five times (for different genes or within a gene family). Only the most frequent reticulations (top 10% quantile counts) are shown. Arrows indicate where transfers are highly skewed in one direction (> 50% of proportional difference). Branch lengths are not proportional to time, and transfer lines start at the midpoint of a branch but the actual timing was not inferred. (a) Whole grass family, where tips were relabelled with tribes. Note that here, numbers of transfers combine gene trees from all species within a tribe. (b) Maize tribe, Andropogoneae. (c) Bamboos, Bambusoideae. (d) Wheat tribe, Triticeae. See also detailed plots in Supporting Information Fig. S10.

Reconciliation of gene trees with the species tree under a duplication–transfer–loss model suggests frequent reticulations in the grass family (Fig. 2, see also detailed plots in Fig. S10). The tribe‐level reconciliation for the whole tree suggests reticulation early in the history of the grasses, involving the branch leading to the large crown group, the BOP–PACMAD clade (Fig. 2a). At this level of analysis, the most frequent reticulations primarily occurred in one direction (see arrows in Fig. 2a). Within Bambusoideae, the inferred transfers for both woody bamboo tribes, Arundinarieae and Bambuseae, reflect the allopolyploid origins of their subgenomes (Triplett *et al*., 2014; Guo *et al*., 2019; Chalopin *et al*., 2021; Ma *et al*., 2024). Note that in this tribe‐level analysis, the number of transfers combine gene trees from all species within a tribe, that is high numbers could be driven either by a few genes or a few (or a single) species. We interpret transfers inferred from ancestors to descendants as transfers to a lineage that is now extinct (or not sampled in our tree) but descended from the same common ancestor. Note that apparent reticulations, especially between lineages branching in short succession, could instead be due to incomplete lineage sorting, which is not modelled by GeneRax. However, the frequency of inferred transfers between more distant lineages does support reticulation, in addition to incomplete lineage sorting.

Reconciliations at the species level (Fig. 2b–d) also support frequent reticulation. In Andropogoneae and Bambusoideae, the most frequent reticulations are not between deeper branches but within particular clades, such as within Andropogoninae, the temperate woody bamboos (Arundinarieae), and, within the paleotropical woody bamboos (Bambuseae), the Malagasy Hickeliinae bamboos and the *Bambusa–Dendrocalamus–Gigantochloa* complex. In Triticeae, reticulation is frequent across the tribe. The assembled genome of the known allohexaploid *Thinopyrum intermedium* accounts for a large proportion of the highly supported transfers in Triticeae (species in bold in Fig. 2d). The origin of *Pascopyrum smithii* from past hybridisation between *Elymus* and *Leymus* (Dewey, 1975), and the origins of bread wheat, *Triticum aestivum*, from *Aegilops* ancestors are also evident.

### Nuclear–plastome tree comparison

**Fig. 3:**
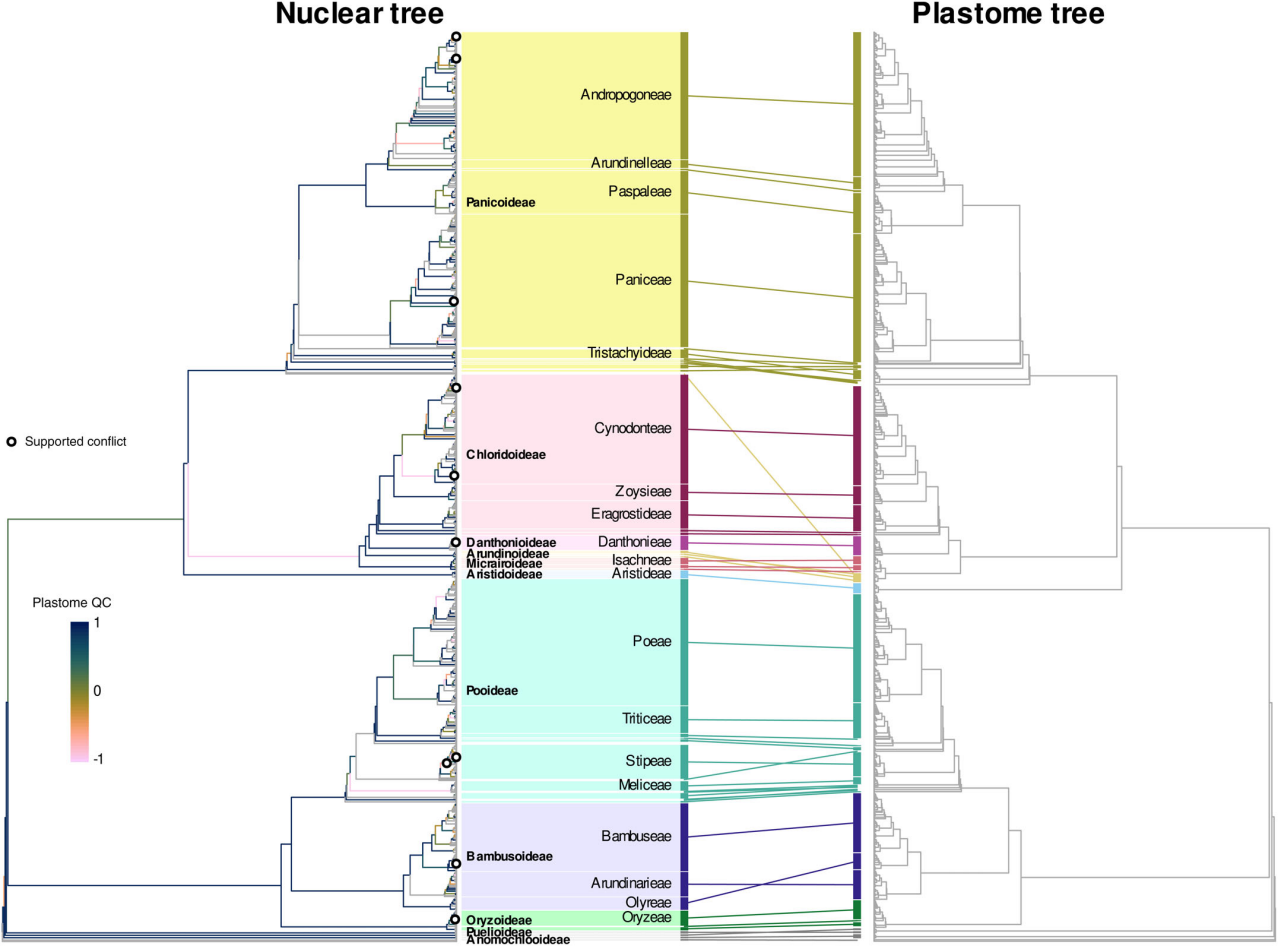
Comparison of nuclear and plastome topologies for the Poaceae. The 1153‐tip nuclear tree is shown on the left, the 910‐tip plastome tree on the right. Plastome support from bootstrap trees (Quartet Concordance, QC) was summarised for branches present in both trees (751 shared species). Grey branches in the nuclear tree had no equivalent for comparison in the plastome tree. Open circles indicate strong signals of conflict, that is high support in the nuclear tree (nuclear QC > 0.2) and high support for an alternative configuration in the plastome tree (plastome QC < −0.2). Tribes are matched between the two trees, and larger tribes are labelled for orientation. See also detailed version of the plastome tree in Supporting Information Fig. S11.

Nuclear–plastome conflict is rare across the grass phylogeny. We inferred a plastome tree for 910 accessions, representing 893 species, 508 genera and all tribes except Ampelodesmeae and Steyermarkochloeae (Fig. 3; see also detailed plot broken down into subclades in Fig. S11). Of these, 751 species, 53 tribes and 478 genera were also present in the nuclear tree and their relationships are compared between both trees (Fig. 3). Most branches in the nuclear tree were also highly supported by plastome data (74% with plastome QC > 0.2). Only 10 branches showed strong signals of conflict, that is they were highly supported in the nuclear tree (nuclear QC > 0.2) and had strong support for an alternative configuration in the plastome tree (plastome QC < −0.2), all of them at shallow levels (open circles in Fig. 3). Nuclear and plastome QC values were positively correlated (*t* = 9.47, *P* < 0.001; Pearson's correlation test, two‐sided), i.e. branches that show a different configuration in the plastome tree tend to be those with high intra‐nuclear conflict.

When directly comparing the positions of clades at subfamily to tribe level, differences are evident in some cases but mostly not strongly supported. The Puelioideae genera *Guaduella* and *Puelia* are sister taxa in the plastome tree (Puelioideae) but not in the nuclear tree (paraphyletic Puelioideae). Note that there is high concordance among the nuclear gene trees grouping Puelia with BOP + PACMAD (QC = 0.6), but conflict in the gene trees grouping *Guaduella* sister to this group (QC < 0.05), so that there is no strongly supported nuclear–plastome conflict in this case. Arundinoideae and Micrairoideae are sisters in the plastome tree but paraphyletic in the nuclear tree, with high‐gene tree incongruence. A striking difference was found in the position of *Styppeiochloa hitchcockii*, placed in Arundinoideae in classifications and in the plastome tree, but as sister to Panicoideae in the nuclear tree, based on the same target capture sample. In Pooideae, tribe Diarrheneae, although monophyletic in plastome trees (Gallaher *et al*., 2022), is polyphyletic in the nuclear tree, as its two genera *Diarrhena* and *Neomolinia* align in different clades; our plastome tree does not include *Neomolinia*. Triticeae plastomes appear to be paraphyletic with regard to Bromeae, as described previously (Bernhardt *et al*., 2017), while Triticeae is monophyletic in the nuclear tree. Finally, the nuclear tree grouped the two woody bamboo tribes, Arundinarieae and Bambuseae, which have distinct allopolyploid origins (Triplett *et al*., 2014; Guo *et al*., 2019; Chalopin *et al*., 2021; Ma *et al*., 2024), while in the plastid tree they are paraphyletic with regard to the herbaceous bamboos, Olyreae (Sungkaew *et al*., 2008). Below tribe level (see detailed tree in Fig. S11), the nuclear tree confirms previous studies in finding the C_4_‐photosynthetic subtribe Anthephorinae (Paniceae) sister to the C_4_ MCP clade of Melinidinae, Cenchrinae and Panicinae (Washburn *et al*., 2015, 2017; Huang *et al*., 2022), but with strong gene tree incongruence between the subtribes or Paniceae. The chloroplast lineage of Anthephorinae is sister to the rest of Paniceae as in previous studies (GPWG II, 2012; Washburn *et al*., 2017; Saarela *et al*., 2018; Gallaher *et al*., 2022). Further differences in the branching order of subtribes are found in the tribes Arundinarieae (temperate woody bamboos), Bambuseae (tropical woody bamboos), Paspaleae, and Poeae.

## Discussion

### Nuclear phylogenomic data support relationships of current subfamilies and tribes despite gene tree incongruence

We show that the nuclear genome topology of the grass family overall supports the monophyly of accepted subfamilies and tribes (Kellogg, 2015; Soreng *et al*., 2022), despite the prevalence of gene tree conflict across the grass phylogeny. The subfamily‐ to tribe‐level classification of the grasses has proven remarkably stable over previous community‐wide phylogenetic efforts (GPWG, 2001; GPWG II, 2012). Our nuclear phylogenomic analysis further substantiates this framework, building on previous work to provide the largest nuclear phylogenomic sampling to date, with 79% of grass genera and all but two small tribes. Such sampling has previously been a considerable challenge in such a species‐rich family. This phylogeny will help clarify generic limits and guide the search for useful genes and traits in wild relatives of cereal, forage, biofuel and turf crops.

Some taxonomic realignments will be necessary, despite the overall consistency with previous work. In addition, the placement of a few taxa will need to be validated by additional sequences or samples (Table 1). These may represent cases of biological interest (e.g. true reticulations) but our current data cannot entirely rule out possible technical artefacts. More taxonomic mismatches will require attention at the subtribe level. Using the sequence data of Huang *et al*. (2022) we were able to reproduce their results suggesting paraphyly of subfamily Puelioideae (*Guaduella* and *Puelia*) in the nuclear tree; there is, however, high‐nuclear incongruence and this result was not tested with independent plant samples. If the cyto‐nuclear conflict continues to be supported, the well‐supported monophyly of the group in the plastid phylogeny would suggest an introgression event in the early history of the grasses. Future morphological studies are needed to determine whether there are characters that support either monophyly or paraphyly of Puelioideae, one of the least well known of the grass subfamilies. More generally, as nuclear genome‐scale data continue to accumulate, the grass taxonomic community will have to decide whether the nuclear genome, ultimately underlying most phenotypic characters, should dictate taxonomy in case of conflicting signals. In the bamboos, the nuclear topology better reflected morphological differences than the plastome phylogeny in previous work (Wang *et al*., 2017). In Paniceae, the position of Anthephorinae sister to Melinidinae–Cenchrinae–Panicinae would be in line with a common origin of C_4_ photosynthesis in the combined clade (Washburn *et al*., 2015), with two separate plastome sources. We refer taxonomic and nomenclatural changes to further studies by specialists of the relevant grass subgroups.

Nuclear–plastome discordance is rare between higher taxonomic levels in the grasses. In the large PACMAD clade, we confirm previous nuclear (Bianconi *et al*., 2020; Huang *et al*., 2022) and plastome studies (GPWG II, 2012) in finding Aristidoideae sister to the other five subfamilies. However, there is high‐nuclear gene tree incongruence, while the plastome PACMAD relationships are highly resolved. More recent plastome studies (Saarela *et al*., 2018; Duvall *et al*., 2020; Gallaher *et al*., 2022) suggested this position might be artifactual and favoured a ‘panicoid sister’ hypothesis. Arundinoideae and Micrairoideae subfamilies do not form a clade as in the plastome tree, a result also found in the nuclear analysis of Huang *et al*. (2022). There clearly is a concentration of gene tree conflict at the base of PACMAD, a period of rapid grass diversification (Christin *et al*., 2014). This suggests the split of the PACMAD subfamilies could represent a hard polytomy, analogous to deep radiations in groups such as Amaranthaceae (Morales‐Briones *et al*., 2021b), Fabaceae (Koenen *et al*., 2021), or neoavian birds (Suh, 2016). Plastome lineages may have sorted more rapidly due to geographically more limited seed compared to pollen dispersal. Across angiosperms, episodes of rapid diversification are correlated with higher conflict among gene trees (Guo *et al*., 2023; Zuntini *et al*., 2024). Further investigation of this relationship for the grasses could build on the dataset we compiled here but will require tackling the complex issue of time calibration in grasses (to be described later).

### Incomplete lineage sorting and reticulation have been frequent in the grass family

Incomplete lineage sorting (ILS) may explain much of the gene tree incongruence in our data. Grasses often have very large ranges and population sizes (Linder *et al*., 2018), which will favour ILS at speciation. Frequent ILS would imply that species delimitation based on only a few markers may be unreliable in the grasses, and paraphyletic species common. However, it is unclear why we find little support for introgression or hybridisation, although we know it must be frequent: *c*. 45–80% of grasses are polyploid (Stebbins, 1985; DeWet, 1986), with an appreciable proportion of those being allopolyploid, that is hybrids. Unequal paralogue recovery and a species sampling not dense enough could mean that signals of hybridisation get blurred in the type of data we used and produce gene tree distributions similar to those expected under ILS.

Nevertheless, gene tree–species tree reconciliation does illustrate the potential scale of nontree‐like phylogenetic structure. It needs to be followed by more in‐depth analyses with phased genomic data that can clearly distinguish reticulation from ILS on closely related branches, and different modes of reticulation from each other. The methods used here are not designed to detect allopolyploidy but are still able to identify frequent reticulation events. The actual modes of reticulation in grasses certainly need more study, as we cannot distinguish here between introgression and hybrid speciation. Recent work also demonstrated the frequency of lateral gene transfers of large blocks in the genomes of *Alloteropsis semialata* (Dunning *et al*., 2019; Raimondeau *et al*., 2023) and other grass species (Hibdige *et al*., 2021). Contamination and gene tree errors can potentially obscure patterns in short‐read data as we included in our analysis, but encouragingly, we retrieved known patterns such as the mosaic origins of the *Thinopyrum intermedium* genome (Mahelka *et al*., 2011). This suggests that reduced‐representation nuclear datasets do retain signals of reticulation.

Our analysis offers a glimpse of how the accumulation of assembled genome data for grasses beyond model and crop species could foster research on reticulation, particularly in three areas. First, clarifying where apparent reticulate relationships may actually stem from differential retention of homologs after whole‐genome duplication. For example, the reticulations we inferred at the base of the BOP–PACMAD clade, the large crown radiation of grasses, could potentially be remnants from the rho whole‐genome duplication event at the stem of Poaceae (McKain *et al*., 2016; Zhang *et al*., 2024). Second, correlating reticulation frequency with ecological and morphological predictors to identify the physical mechanisms of lateral transfers, which remain speculative (Pereira *et al*., 2023). Third, using synteny information to identify the precise locations and origins of functional variation, potentially using new deep learning approaches for identifying introgression (Zhang *et al*., 2023). New crops, such as *Thinopyrum intermedium* (intermediate wheatgrass or kernza) with its mosaic genome and its potential as perennial cereal or genetic resource (Baker *et al*., 2020), are certainly prime candidates for such research. However, given the frequency of allopolyploidisation, and if lateral transfers are as frequent as recent work suggests (Hibdige *et al*., 2021), the grass family as a whole may well constitute a ‘single genetic system’ (Freeling, 2001; Mascher *et al*., 2024) or higher‐level ‘pangenome’ (Dunning *et al*., 2019). The lateral recruitment, across 20 million years of divergence, of key genes for C_4_ photosynthesis in panicoid grasses (Christin *et al*., 2012), illustrates this point. Species‐level sampling including the more distant relatives of crops is therefore needed to access the entire genetic diversity potentially available for future sustainable agriculture.

### Towards a complete grass tree of life

Our community effort resulted in the most comprehensive nuclear phylogenomic tree for the grass family to date, including 1133 species, with 21 genera sequenced for the first time. This tree, and the dataset associated with it, paves the way towards placing all *c*. 11 800 species in the grass tree of life. Already, the International Nucleotide Sequence Database Collaboration hosts sequence data for more than 6200 grass species, as of April 2024. The comprehensive phylogenomic backbone we provide here could provide a basis for assembling these shorter sequences into a grass supertree for analyses of trait evolution and biogeography, as attempted previously with smaller Poaceae backbones (Spriggs *et al*., 2014; Elliott *et al*., 2024).

We show that the Angiosperms353 gene set can be successfully used to anchor different types of genomic datasets, including unenriched Illumina sequence data. Sequencing depth and paralog recovery obviously vary across such different datasets, which needs to be taken into account, for example in future studies of whole‐genome duplications and events of auto‐ and allopolyploidy (Thomas *et al*., 2017; Morales‐Briones *et al*., 2021a; Rothfels, 2023) or large‐scale gene duplications that preceded major innovations like cold tolerance (Schubert *et al*., 2020; Zhang *et al*., 2022). The extent to which paralog‐aware methods of species tree inference are robust to unaccounted paralogs in large datasets has yet to be evaluated, but this problem should decrease in importance as more high‐coverage datasets become available. The improved Angiosperms353 reference set constructed here for the grasses will facilitate the inclusion of previously unsequenced grass species. This target capture approach allows in particular sequencing degraded DNA from herbarium specimens in a cost‐efficient manner and thus filling the remaining gaps of the grass tree of life, even where there are logistical barriers to obtaining high‐molecular weight DNA for full‐genome sequencing.

The timeline of grass evolution continues to be a matter of debate, with recent studies suggesting a mid‐Cretaceous origin for the grasses (Gallaher *et al*., 2022; Huang *et al*., 2022) and thus supporting earlier suggestions based on phytolith fossils (Prasad *et al*., 2005, 2011). However, such age estimates hinge on several factors (Christin *et al*., 2014), such as the placement of phytolith fossils, appropriate modelling of rate correlation, and the upper bound set by the age of flowering plants, which itself remains unclear and fraught with methodological challenges (Brown & Smith, 2018; Silvestro *et al*., 2021; Sauquet *et al*., 2022; Carruthers & Scotland, 2023). The nuclear dataset we provide here, along with recent advances in grass phytolith classification (Gallaher *et al*., 2020) as well as a better understanding of rate variation across branches (Carruthers *et al*., 2020; Carruthers & Scotland, 2020) and gene tree conflict (Carruthers *et al*., 2022) on divergence time estimation suggest a new comprehensive analysis of grass divergence times as a promising avenue forward.

## Competing interests

None declared.

## Author contributions

WJB, MEB, P‐AC, LTD, JH, EAK, RJS and MSV conceptualised the study. MDB, RLB, GB, MEB, P‐AC, PC, DMC, GD, LTD, MRD, SD, SZF, SF, JH, TRH, WH, RWJ, EAK, JMK, XL, OM, TGBM, MFM‐A, DJM, JR, LS, RJS, MSV, MW, CADW, MDX, LZ and FOZ curated the data. MEB and JH performed formal analysis. WJB, GB, P‐AC, JTC, DMC, FF, EAK, LZ and AZ acquired funding. MEB, JTC, JH and IL performed investigation. WJB, MEB, P‐AC, LTD, JH, AMH, EAK, RJS, MSV and ARZ developed methodology. WJB, MEB, P‐AC, JH and MSV had administrative responsibility in the project. WA, MDB, RLB, JL Bennetzen, JL Birch, GB, PC, WC, MC, LGC, JCAC, DMC, GD, MRD, SD, AEF, SF, FF, LJG, TH, TRH, C‐HH, RWJ, EAK, CJK, JMK, IL, RL, D‐ZL, J‐XL, XL, QWRL, HM, TDM, OM, MRM, TGBM, DJM, OPN, GEO, PMP, RAR, JR, JMS, LS, NWS, RJS, MSMS, EJT, PT, GAV, MSV, NGW, JDW, TW, MW, CADW, MDX, NX, LZ and FOZ provided resources. MEB and JH developed software code. WJB, P‐AC, EAK, RJS and MSV supervised the project. GB, MEB, LTD, JH, EAK, JMK, D‐ZL, J‐XL, HM, RJS, MSMS, MSV and ARZ validated the results. RLB, MEB and JH visualised the data. MEB and JH wrote the original manuscript. WA, WJB, MDB, RLB, JB, GB, MEB, P‐AC, WC, PC, LGC, DMC, LTD, AEF, FF, JH, TRH, WH, AMH, RWJ, EAK, JMK, IL, D‐ZL, HM, MRM, TGBM, DJM, JMS, NWS, RJS, MSMS, MSV, NGW, JDW, MW, CADW, LZ and AZ reviewed and edited the manuscript.

## Supporting information


**Fig. S1** Schematic overview of the custom workflow used for sequence assembly from Illumina shotgun accessions.
**Fig. S2** Nuclear gene recovery and sequence completeness.
**Fig. S3** Paralog recovery across data types.
**Fig. S4** Effect of sequencing depth on the recovery of paralogs in shotgun accessions.
**Fig. S5** Overall paralog recovery across accessions.
**Fig. S6** Test of the custom assembly workflow on full‐genome sequences.
**Fig. S7** Test of the custom assembly workflow on full‐genome sequences – copy number recall.
**Fig. S8** Detailed version of the multispecies coalescent nuclear species tree.
**Fig. S9** Nuclear species tree stability under different data filtering strategies.
**Fig. S10** Detailed plots of the reticulations inferred with gene tree–species tree reconciliation.
**Fig. S11** Detailed version of the plastome tree.
**Methods S1** DNA isolation, library preparation, sequencing and curation of the grass‐specific Angiosperms353 reference dataset.

**Table S1** Nuclear alignment summary statistics.
**Table S2** Number of gene copies (paralogues) recovered per accession and gene.
**Table S3** HybPiper nuclear gene assembly statistics.
**Table S4** Custom pipeline assembly statistics for shotgun accession.
Please note: Wiley is not responsible for the content or functionality of any Supporting Information supplied by the authors. Any queries (other than missing material) should be directed to the *New Phytologist* Central Office.

## Data Availability

Data used and produced in this study, including metadata for all accessions, gene alignments and phylogenetic trees are available in an open Zenodo repository (doi: 10.5281/zenodo.10996136). New short‐read data and plastome assemblies are available via the International Sequence Database Collaboration (INSDC), BioProject no. PRJEB79360.
